# Spatial protein redistribution: wandering but not lost

**DOI:** 10.1007/s00018-025-05803-9

**Published:** 2025-08-21

**Authors:** Faiza Amterat Abu Abayed, Laila Abu Madegam, Ayelet Gilad, Gal Twito, Suad Sheikh Suliman, Suma Biadsy, Aeid Igbaria

**Affiliations:** Department of Life Sciences, Ben-Gurion University of the Negev, Beer Sheva, Israel

**Keywords:** Endoplasmic reticulum, Proteostasis, Spatial proteostasis, ER to cytosol signaling (ERCYS)

## Abstract

Interorganellar spatial redistribution of proteins represents a critical yet underexplored facet of eukaryotic cell biology. This dynamic aspect of proteostasis allows proteins to acquire novel functions based on their subcellular localization, enabling the cell to adapt to both physiological and pathological challenges. Such spatial reprogramming is especially pronounced under stress conditions, including those associated with cancer, neurodegenerative diseases and viral infection, where widespread remodeling of the proteome facilitates survival and adaptation. Despite increasing appreciation of its biological significance, the molecular mechanisms underlying protein relocalization, as well as the functional outcomes of interorganellar trafficking, remain incompletely understood. This review highlights recent advances in the field, with a particular focus on the redistribution of proteins from the endoplasmic reticulum (ER) to other organelles. We provide a detailed examination of a recently characterized mechanism by which cytosolic and ER-resident chaperones and cochaperones mediate the extraction of proteins from the ER into the cytosol. Furthermore, we explore the fate of these relocalized proteins, the mechanistic underpinnings of their trafficking, and how this process compares with other modes of intracellular protein redistribution. Understanding these pathways offers valuable insights into fundamental cell biology and unveils new avenues for therapeutic intervention.

## Introduction

Prokaryotic and eukaryotic cells share fundamental features, including a plasma membrane and ribosomes that facilitate protein synthesis by translating genetic information from DNA to functional proteins. A key distinction, however, lies in their intracellular organization. Eukaryotic cells have evolved diverse membrane-bound and membraneless organelles within the cytoplasm, enabling a higher degree of spatial and functional compartmentalization. Membrane-bound organelles, in particular, are thought to carry out specialized functions relatively autonomously, distinct from the activities of other cellular compartments. This increased organizational complexity is a defining feature of eukaryotic cells and is essential for supporting the regulation, coordination, and integration of diverse cellular processes [1].

The endoplasmic reticulum (ER) is the largest organelle in the cell and plays a central role in coordinating intracellular and extracellular functions, including protein synthesis, folding, and secretion [2–4]. Under conditions of cellular stress, the ER experiences an accumulation of misfolded or unfolded proteins, leading to a state known as ER stress. To alleviate this burden, cells activate multiple adaptive mechanisms to reduce protein load within the ER. These include mRNA degradation of ER-targeted transcripts, global translational attenuation, ER-phagy (selective autophagic degradation of the ER), and proteasomal clearance of misfolded proteins [5–9]. Recently, an additional layer of regulation has emerged: the redistribution of ER client proteins to other cellular compartments to relieve ER load. These spatial redistributions have been observed across various human diseases and are now being linked to functional roles outside the ER, implicating them in broader cellular signaling pathways [10–14]. Notably, many of these non-ER localizations, previously dismissed as experimental contaminants or with no explanation, underscore the need to reevaluate organelle-specific proteomics in light of dynamic stress responses [15–18].

Protein redistribution across subcellular compartments is not limited to the ER; numerous proteins have been reported to relocate to alternative organelles through mechanisms such as dual targeting or less well-characterized, non-canonical trafficking pathways [19–21]. Unlike classical dual targeting, where proteins possess intrinsic targeting signals guiding them to multiple destinations, redistribution likely involves stress- or signal-induced relocalization mechanisms that actively move a protein from one compartment to another. Notably, upon relocalization, many of these proteins acquire novel, context-dependent functions, distinct from those executed in their original compartments. Understanding such spatial interorganellar protein redistribution offers insight into how a single gene product can be repurposed to fulfill functionally distinct roles depending on its localization. This redistribution strategy is both economical and adaptive, allowing cells to repurpose existing proteins in response to changing conditions. However, it also introduces a higher level of complexity to the regulation of cellular proteostasis, challenging traditional compartment-based models of protein function.

In this review, we explore the diverse mechanisms of protein redistribution in eukaryotic cells, with an emphasis on distinguishing their underlying processes and biological significance. Particular attention is given to a recently described mechanism termed ER-to-Cytosol Signaling (ERCYS), in which ER-associated proteins are selectively rerouted to the cytosol via a chaperone-mediated process. This form of protein relocalization is associated with novel, context-specific gain-of-function activities, many of which remain poorly understood. By examining ERCYS alongside other interorganellar redistribution pathways, we aim to highlight its potential as a key regulator of cellular plasticity and stress adaptation.

### The importance of protein compartmentalization in eukaryotic cells

The internal organization and compartmentalization of eukaryotic cells are essential for cellular functionality and viability. These specialized microenvironments facilitate cellular biochemical reactions by ensuring precise concentrations of enzymes and substrates and by regulating factors such as pH, redox potential, and osmolarity (Dacks et al., 2016). First shown by *George Palade*, this organizational principle underlies the presence of membrane-bound organelles such as the ER, nucleus, mitochondria, and Golgi apparatus [22–25]. These membranes are composed of lipid bilayers, similar to the outer membrane but not identical. Each organelle performs distinct roles in metabolism, signaling, and cellular homeostasis, while protecting sensitive components from undesired by-products. For instance, the ER maintains a highly oxidizing environment to facilitate oxidative protein folding; lysosomes maintain an acidic lumen for degradation by acid hydrolases; and the mitochondria utilize electrochemical gradients across their membranes to drive ATP synthesis. Additionally, compartmentalization enables dynamic regulation of cellular processes through the spatial redistribution of proteins and signaling complexes [26].

Recent discoveries have expanded this concept to include membraneless structures such as multi-enzyme complexes and biomolecular condensates, which mimic some functions of classical organelles by organizing metabolic reactions in space and time. Perturbations in either membrane-bound or membraneless compartments can disrupt proteostasis and metabolic integrity, underscoring their critical roles in maintaining cellular function and preventing disease [27–31]. The evolution of such organelles marked a pivotal advancement in eukaryotic biology, greatly enhancing the ability of cells to adapt and respond to environmental fluctuations.

The first step in protein compartmentation is to direct the synthesized proteins/polypeptides to the proper organelle. This process relies on sequences essential for translocating the synthesized polypeptides/proteins into the home organelle by organelle-specific import machinery that can recognize those sequences. Hence, eukaryotic cells developed precise protein targeting and retention systems to maintain functional organization and ensure that proteins are delivered to and retained within the correct subcellular compartments [26, 32]. These systems rely on a combination of amino acid signal sequences, chaperones, and receptor-mediated transport mechanisms that recognize and direct proteins during or after translation [33, 34].

Most mitochondrial proteins are encoded by nuclear DNA and are translated on the cytosolic ribosomes. Those mitochondria-localized proteins possess a mitochondrial localization signal (MLS), sometimes termed mitochondria targeting sequence (MTS). This signal is a short peptide of 20–70 amino acids with common characteristics: a lack of negatively charged residues and an arginine-rich sequence [35, 36]. The translated proteins containing the MTS/MLS are then directed to the mitochondrial surface and are recognized by the translocase of the outer mitochondrial membrane (TOM). Once there, those proteins then bind to other mitochondrial import machinery, including the translocase of the inner mitochondrial membrane (TIM) and other chaperone complexes, to facilitate their import into specific mitochondrial compartments [37–39].

Peroxisomal proteins are targeted to the peroxisome matrix by peroxisomal targeting signal type 1 (PTS1) or peroxisomal targeting signal type 2 (PTS2). PTS1 is a C-terminal extension recognized by the cytosolic receptor peroxisomal biogenesis factor 5 (Pex5) and Pex13/14. PST2 is a less common N-terminal nonapeptide that is recognized by Peroxisomal biogenesis factor 7(PEX7) [40, 41]. On the other hand, nuclear proteins often contain nuclear localization signals (NLS), short peptide sequences that help direct proteins from the cytoplasm into the nucleus. NLS is rich in lysine and arginine and is essential for cargo proteins to traverse the nuclear pore complex via members of the importin superfamily [42, 43].

### The endoplasmic reticulum: central hub for protein folding and trafficking

The ER is the largest organelle within the eukaryotic cell, and it forms an extensive network of flattened sacs that maintain physical connections with multiple organelles, including the nucleus, mitochondria, and peroxisomes. Structurally, the ER is divided into two distinct regions: the rough ER, characterized by the presence of ribosomes on its cytosolic surface and specialized in protein synthesis, and the smooth ER, which lacks ribosomes and is primarily involved in lipid biosynthesis, calcium storage, and detoxification processes. The ER functions as a central hub for the folding and maturation of proteins in the secretory pathway, as approximately one-third of the eukaryotic proteome undergoes folding within the ER.

Targeting ER-resident proteins and proteins in the secretory pathway to the ER follows the same targeting code as that of other organelles. ER proteins possess an N-terminal signal sequence that directs them to the ER lumen [44]. This is a highly variable short sequence composed of up to 30 positively charged amino acids that contain a core of hydrophobic residues [3, 33, 34]. ER-resident proteins are translated by ribosomes positioned on the cytosolic side of the ER membrane. Upon translation, the signal sequence is recognized by the signal recognition particle (SRP), a ribonucleoprotein complex on the ER membrane that facilitates pre-protein insertion into the ER and protects the translated mRNA from degradation [33, 34]. These nascent polypeptides undergo co-translational translocation into the ER lumen through a gate-like multi-protein complex called the translocon. The translocon consists of different proteins, including the SRP and the SEC family member protein SEC61—the most important translocon component that forms the channel. SEC61 allows proteins to be imported into the ER and provides a direct pathway to the lipid bilayer, allowing nascent membrane proteins to be incorporated into the ER membrane [45, 46]. Once on the channel, the ER-resident HSP70 family member BIP/GRP78 (Kar2p in yeast) acts as a molecular ratchet. This activity of BIP ensures the forward movement of the translated proteins into the ER by pulling them inside. Then, the signal peptide is enzymatically cleaved at positions −1 and −2 [32, 33, 47, 48]. Soluble ER-resident proteins contain a conserved retention signal motif Lys-Asp-Glu-Leu (KDEL) at their C-terminus, recognized by the KDEL receptor. The KDEL receptors are seven-transmembrane domain proteins that mediate the retrieval of ER-resident proteins from the Golgi apparatus back to the ER [49–51].

In addition to specific targeting and retention signals, some proteins are retained within organelles through stable protein–protein interactions or membrane anchoring. For example, Calreticulin, a calcium-binding molecular chaperone of the ER, is retained within the ER lumen by forming complexes with other ER-resident proteins and through its affinity for the ER luminal environment, including calcium and pH conditions that shape its structural confirmation [52]. Similarly, Cytochrome c, a key electron carrier in the mitochondrial electron transport chain, is retained in the intermembrane space of mitochondria by electrostatic and hydrophobic interactions with cardiolipin, a mitochondria-specific phospholipid located on the inner mitochondrial membrane [53].

### ER stress signaling and the stress response for unfolded proteins

Correctly folded proteins are transported from the ER to the Golgi apparatus for further sorting and secretion. In contrast, misfolded proteins accumulate within the ER, triggering a condition known as ER stress. ER stress arises when the demand for protein folding exceeds the capacity of ER-resident chaperones and folding enzymes, resulting in the buildup of unfolded or misfolded proteins. To mitigate this stress and restore proteostasis, cells activate the unfolded protein response (UPR)—a conserved signaling pathway designed to enhance the ER’s folding capacity, reduce the influx of new proteins, and promote the degradation of misfolded substrates [3, 8].

Three sensors control the UPR: Inositol Requiring Enzyme-1 (IRE1), Protein kinase R (PKR)-like Endoplasmic Reticulum Kinase (PERK), and Activating Transcription Factor 6 (ATF6) [54–60]. IRE1 is a transmembrane kinase and endoribonuclease located in the ER and serves as a key sensor and effector of the UPR. Upon activation, IRE1 catalyzes the unconventional splicing of X-box binding protein 1 (XBP1) mRNA, producing a spliced form known as XBP1s. XBP1s functions as a transcription factor that upregulates a subset of UPR target genes, thereby enhancing the protein folding capacity of the ER [55, 61–63]. In addition to promoting ER homeostasis by increasing folding capacity, the UPR also reduces the influx of new proteins into the ER to alleviate folding stress. Under sustained or severe ER stress conditions, hyperactivation of IRE1’s RNase domain leads to the degradation of a broad array of mRNAs and microRNA precursors through a Regulated IRE1-Dependent Decay (RIDD). Many of these target transcripts are localized near the ER membrane and encode secretory pathway components [64].

ATF6 activation involves translocation to the Golgi apparatus, where it is cleaved by site-1 and site-2 proteases (S1P and S2P), releasing the active transcription factor ATF6(N). The XBP1/IRE1 axis and the ATF6 arm of the UPR regulate the expression of numerous genes encoding ER-resident chaperones and folding enzymes. This transcriptional program enhances the ER's folding capacity and ER-associated degradation (ERAD) efficiency, thereby facilitating the clearance of misfolded proteins [58]. In parallel, activation of the PERK branch of the UPR leads to phosphorylation of eukaryotic initiation factor 2 alpha (eIF2α), resulting in a transient global reduction in protein translation [57]. This reduction decreases the load of nascent proteins entering the ER while selectively enhancing the translation of mRNAs involved in antioxidant defense and stress adaptation, contributing to increased cell survival [65]. Collectively, these UPR branches coordinate to expand ER folding capacity, reduce protein misfolding, and limit the entry of new substrates into the ER under stress conditions.

### The global protein redistribution as a cellular stress response

Despite the stringent sorting and signaling pathways that direct proteins to their designated organelles and maintain organellar proteostasis, many proteins display dynamic localization rather than remaining confined to a single compartment. The spatial remodeling of proteins is increasingly recognized as an adaptive cellular response to various stressors in eukaryotic systems. Recent studies have highlighted interorganellar redistribution of proteins as a significant hallmark of viral infection [21, 66, 67]. It was shown that extensive spatial remodeling of proteins occurs across multiple organelles, independent of changes in overall protein abundance. Interestingly, the ER exhibited the most pronounced remodeling among all organelles analyzed [21].

One mechanism that regulates such dynamic localization is dual targeting, by which a single gene product is targeted to more than one organelle. This evolutionarily conserved mechanism enhances the functions of proteins without increasing the number of genes encoding for them. Unlike isoforms, which are two different proteins that have the same function, dual localized proteins have the same amino acid sequence but can localize to different organelles and have either similar or different functions. Because of their dual targeting, they can act as tethers between different organelles, such as the ER and peroxisomes or the mitochondria, and can also function as signaling molecules [20, 68, 69].

Dual targeting is a highly regulated process that involves different protein complexes and can be adjusted to meet the requirements of the cells during stress conditions. Dual targeting can be a result of (1) one gene encoding for a protein that has two targeting sequences, (2) two genes encoding for the same protein but only one has a targeting sequence, (3) One gene with a targeting sequence but during translation produces two proteins due to alternative initiation translation, (4) one gene with a targeting sequence but has an alternative transcription initiation, and (5) one gene with a targeting sequence that is removed during maturation by splicing [20].

The transcription factor ATF5 regulates the mitochondrial unfolded protein response (mitoUPR) to restore mitochondrial proteostasis. ATF5 has dual targeting sequences that target the protein to the mitochondria by the MTS or the nucleus by its C-terminal NLS. Under normal conditions, the MTS directs ATFS-1 to the mitochondria through the HAF-1 import channel, which is then degraded by the CLpP protease [70]. During stress, HAF-1 is blocked, which leads to the cytosolic accumulation of ATF5, favoring its translocation and accumulation in the nucleus where it forms a complex with the small ubiquitin-like protein, UBL-5, and the transcription factor DVE-1 [8–10]. This active complex mediates the transcriptional activation of genes encoding for proteases and chaperones associated with the mitoUPR [70].

**Fig. 1:**
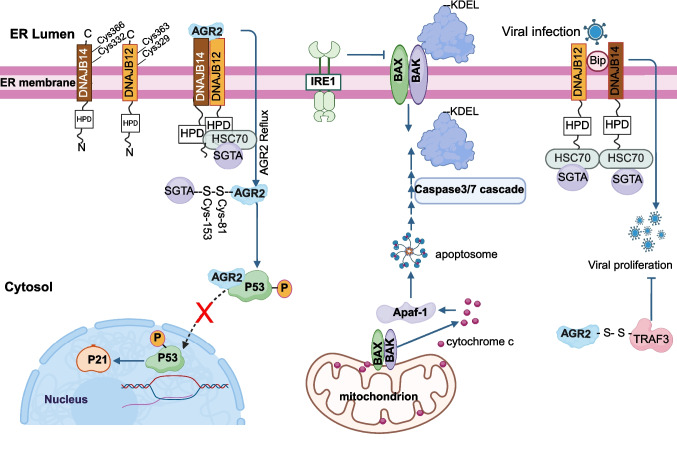
Mechanisms of Endoplasmic Reticulum (ER) Protein Escape. DNAJB12 and DNAJB14 oligomerize and bind to refluxed ER proteins, exemplified by AGR2. These J-domain proteins subsequently recruit HSC70 and its co-chaperone SGTA, facilitating the extraction of bound ER proteins into the cytosol. This process mirrors the mechanism described for the cytosolic translocation of non-enveloped viruses. Once in the cytosol, AGR2 interacts with SGTA and is directed to bind wild-type p53 (wt-p53), resulting in its functional inhibition. During viral infection, AGR2 also forms a redox-dependent complex with TRAF3. Alternatively, ER-resident proteins may escape through BAX/BAK channels localized to the ER membrane during apoptosis, via an IRE1-dependent pathway reminiscent of cytochrome c release from mitochondri

Dual targeting is not the only mechanism to control interorganellar spatial redistribution. Though. A well-characterized gain of function of a protein outside its home organelle is cytochrome c. In response to apoptotic stimuli, cytochrome c is released into the cytosol following the permeabilization of the mitochondrial outer membrane. This process is typically mediated by pro-apoptotic Bcl-2 family members, such as BAX and BAK, which oligomerize to form pores in the outer membrane. Once in the cytosol, cytochrome c interacts with apoptotic protease-activating factor-1 (Apaf-1) to form the apoptosome, activating caspase cascades and ultimately driving programmed cell death [71]. Thus, this redistribution shifts cytochrome c from an essential and vital protein to a lethal one (Fig. 1).

Furthermore, Cysteine cathepsins are lysosomal proteases primarily involved in proteolytic degradation within the lysosomal compartment. However, upon activation of the extrinsic apoptotic pathway, cathepsin B (CatB) has been shown to relocate to the cytosol, contributing to the execution of programmed cell death by promoting the cross-activation of caspase-9 by caspase-8 [72, 73]. The relocalization of ATF5, cytochrome c, and CatB underscores the functional versatility of proteins that adapt their functions in response to changes in their location, regardless of the redistribution mechanism used for such spatial remodeling.

### Escaping the ER

Several ER proteins have been reported to redistribute both within and outside the cell, though the underlying mechanisms remain unclear. This ER protein redistribution in non-ER locations was shown to be associated with the inhibition or modulation of proapoptotic pathways, including caspase-3/7, HIF1a, STAT3, wt-p53, TRAF3, and others [9–11, 14, 18, 21, 66, 74–83]. These functions differ from the canonical ER-specific chaperone activity of those chaperones and enzymes.

Historically, protein disulfide isomerases (PDIs) were primarily characterized as ER-resident proteins; however, accumulating evidence over the last three decades has demonstrated that PDIs can localize to non-ER compartments, including the cytosol, nucleus, cell surface, and extracellular space [16]. Despite having a well-characterized ER-retention sequence, secreted PDI proteins participate in diverse cellular and pathophysiological processes. For instance, during vascular injury, activated platelets release PDIA3/ERp57 and PDIA6/ERp5, which catalyze the reduction of specific disulfide bonds in inactive plasma vitronectin. These redox modifications promote vitronectin activation and contribute to thrombus formation [84]. In cancer, elevated extracellular levels of various PDIs have been observed in multiple tumor types compared to normal tissues, suggesting a role for PDIs in tumor progression and survival [85]. Moreover, PDIA17/AGR2 was recently shown to be secreted and transmitted to neighboring immune cells to mediate antiviral response through cell–cell communication and TRAF3 inhibition [66].

The mechanisms underlying the ER exit of PDIs are not yet fully elucidated. Evidence suggests that PDIs may be exported through active, Golgi-independent pathways [86]. Another proposed mechanism involves KDELR1-mediated transport, whereby KDELR1 shuttles PDIs to the plasma membrane, potentially facilitating their release near target proteins. The presence of the KDEL sequence may, paradoxically, be required for effective surface localization of PDIs such as PDIA6 [87].

On the other hand, several mechanisms for intracellular and interorganellar redistribution of ER-resident proteins have been proposed. Under stress or pathological conditions, certain proteins exit by ERAD but avoid proteosomal degradation and persist in the cytosol, where they may interact with cytosolic partners or form aggregates. For example, in NGLY1 deficiency, misfolded glycoproteins fail to undergo deglycosylation due to a mutation in the cytosolic NGLY1 (N-Glycanase 1) enzyme, thereby escaping degradation and leading to their accumulation in the cytosol [88]. Similarly, during Hepatitis B virus infection, BiP escorts the viral Precore protein out of the ER. Because the protein has a low lysine content, it avoids ubiquitination and proteasomal degradation, which leads to cytosolic redistribution of BiP [17]. Additional mechanisms, including arginylation and nuclear translocation during viral infection, have also been implicated in BiP relocalization [82]. Moreover, another mechanism suggests that permeabilization of the ER membrane occurs through damage caused by BAX and BAK. This process is controlled by IRE1, which acts as a guardian against the leakage of ER proteins into the cytosol. This mechanism is similar to the exit of cytochrome c from the mitochondria during apoptosis [89].

#### ER to CYtosol signaling (ERCYS)

A recently identified mechanism describes the reflux of ER proteins into the cytosol. This mechanism was initially identified in *Saccharomyces cerevisiae* and shown to be evolutionarily conserved in mammalian cells. This ER stress-induced process results in structural remodeling of the refluxed proteins, including reduced disulfide bonds and, in yeast, cytosolic deamidation of glycoproteins mediated by peptide-N-glycanase (PNGase) activity. In contrast to dual-targeting, ERCYS targets proteins that have already localized to the ER, as shown using the ER-targeted photoconvertible fluorescent reporters [10–14]. Notably, ER-protein reflux operates independently of signal peptide recognition and degradation cues and occurs without overloading the ER translocation machinery during stress [14]. This phenomenon may serve as a protective mechanism to reduce the luminal protein load during stress. Still, it also poses potential consequences for cytosolic proteostasis crisis and signaling disruption, especially if ER-resident chaperones or folding enzymes aberrantly accumulate in the cytosol.

ERCYS is conserved in mammalian cells and impacts a broad range of proteins within the secretory pathway. The refluxed glycoproteins form a distinct functional network in stressed cultured cells and in tumors isolated from human glioblastoma multiform (GBM) patients, suggesting coordinated and biologically relevant roles in cellular homeostasis and stress adaptation. ERCYS is constitutively active in cancer cells and has been observed in freshly resected human tumors, including GBM and colorectal carcinoma (CRC), as well as murine tumor models [11, 12]. Importantly, ERCYS has been shown to function as a non-genetic suppressor of wild-type p53 facilitated by establishing inhibitory interactions of specific ER proteins with pro-apoptotic factors, thereby promoting cell survival. The cytosolic relocalization of the PDI-like proteins such as AGR2 confers a survival advantage to tumor cells frequently exposed to chronic stress by inhibiting proapoptotic pathways. This gain-of-function in the cytosol may serve as an adaptive mechanism by reducing the luminal burden of the ER, thereby complementing classical ER quality control pathways [11, 12]. Thus, ERCYS provides a selective advantage in cancer cells by supporting secretory pathway homeostasis and enabling cytosolic functions that enhance cellular fitness and promote tumor progression. AGR2 represents a critical effector of ERCYS and highlights how ER-derived proteins can be repurposed in the cytosol to support tumor progression by spatial remodeling.

Subcellular relocalization of proteins represents a significant evolutionary adaptation, enabling a single gene product—once transcribed and translated—to perform distinct functions depending on its intracellular location. This form of non-genetic functional diversification is particularly relevant in the context of disease, including cancer, where it may contribute to chemoresistance by enabling stress-responsive proteome remodeling. Moreover, this spatial repurposing of proteins is exploited by various viruses, which hijack conserved host trafficking mechanisms to escape the ER [90]. In doing so, they utilize shared chaperone networks and protein complexes from both the ER and cytosol, highlighting how this evolutionarily conserved process can be co-opted to support viral replication and immune evasion.

#### Molecular ratchet in A sharp U-Turn

Mechanistically, ERCYS is distinct from classical ERAD and pre-emptive quality control (pre-QC) pathways, as it does not require proteasomal degradation nor involve the canonical ERAD machinery [10, 14]. Reflux operates independently from E3 ligase activity and is enhanced when ERAD components are impaired, indicating a parallel operational mechanism. Unlike pre-QC and ERAD, where misfolded or unassembled proteins are retrotranslocated to the cytosol in a non-native state for degradation, ERCYS involves the selective reflux of fully translocated ER-resident proteins that retain structural integrity and functional potential in the cytosol. Instead, ERCYS appears to function through a chaperone-dependent, vectorial mechanism reminiscent of a reversed molecular ratchet, requiring coordinated action between ER-resident and cytosolic chaperones to facilitate the directional relocalization of proteins from the ER lumen to the cytosol.

In *Saccharomyces cerevisiae*, the ER-resident Hsp40 co-chaperone Hlj1 is necessary and sufficient to drive protein reflux from the ER. During ER stress, Hlj1-dependent interactions facilitate the engagement of the cytosolic Hsp110 chaperone, Sse1, which functions as a holdase and nucleotide exchange factor. Sse1 binds to refluxed ER clients in a manner dependent on Hlj1, underscoring a tightly regulated chaperone relay [10, 14]. This process constitutes a chaperone-mediated"ratcheting"mechanism that operates reversely to the well-characterized vectorial translocation of proteins into the ER, as described in Kar2-dependent translocation studies by T. Rapaport and colleagues [91].

In mammalian cells, the ER membrane-localized co-chaperones DNAJB12, DNAJB14, and the cytosolic HSC70-cochaperone SGTA are key mediators of ER-to-cytosol reflux during proteotoxic stress. DNAJB12 and DNAJB14 are required for efficient reflux of ER proteins; however, DNAJB12 is uniquely necessary and sufficient to activate the ERCYS pathway and suppress wild-type p53 function. Silencing of SGTA disrupts ER protein reflux and restores p53 activity during ER stress, highlighting its functional involvement in this pathway. The chaperone activity of DNAJB12 and DNAJB14 depends on an intact HPD motif within their cytosolically oriented J-domains, suggesting that the HSP70/HSC70 system is essential for ERCYS-mediated retrotranslocation [12, 13]. These findings identify DNAJB12, DNAJB14, and SGTA as critical components linking ER-localized proteins to cytosolic signaling pathways—connections that have long been observed but previously lacked mechanistic clarity (Fig. 1).

Structural modeling using AlphaFold predicts that ERCYS may be regulated through a redox-sensitive mechanism involving critical cysteine residues—Cysteine-81 in AGR2 and Cysteine-153 in SGTA—required for stable complex formation [12]. Additional evidence supporting redox dependence comes from observations that reductive ER stress destabilizes DNAJB12 and DNAJB14 in cancer cells, leading to their proteasomal degradation and the subsequent induction of apoptosis [13, 92]. The degradation of DNAJB12 is redox-regulated and can be prevented by pre-treatment with dimedone, a sulfenic acid–selective probe. The formation of dimedone-labeled P-SOH adducts on DNAJB12 suggests that sulfenylation is a key post-translational modification modulating its stability, consistent with similar regulatory mechanisms described for redox-sensitive ion channels [93]. Furthermore, AlphaFold-based structural predictions indicate the presence of intramolecular disulfide bonds—specifically Cys329–Cys363 in DNAJB12 and Cys332–Cys366 in DNAJB14—supporting a redox-regulated conformational control of these co-chaperones (Fig. 1).

### Physiological and pathological implications of ER protein spatial remodeling

In higher eukaryotes, ER protein reflux may be a pivotal mechanism influencing cell fate decisions under conditions of severe ER stress. Emerging evidence implicates ER proteins in non-ER locations as a significant contributor to the pathophysiology of chronic diseases characterized by persistent ER stress and disrupted proteostasis, including cancer, neurodegeneration, viral infection, and metabolic disorders.

In cancer, the ER is pivotal in supporting tumor cell adaptation to microenvironmental stressors such as hypoxia, nutrient deprivation, and oxidative stress. The UPR is frequently activated under these conditions to maintain proteostasis and promote cell survival [63]. Within this context, ER protein reflux may contribute to the mislocalization of ER-resident chaperones, including GRP78 (BiP), to the cytosol or cell surface, where they engage in oncogenic signaling, immune evasion, and resistance to chemotherapy. Cytosolic GRP78, for example, has been shown to interact with components of the PI3K/Akt pathway, thereby promoting anti-apoptotic signaling [94].

Recent studies have demonstrated that GRP78 can also translocate to the nucleus in cancerous and stressed cells via a defined nuclear localization signal. Once in the nucleus, GRP78 modulates transcriptional programs by interacting with and inhibiting the activity of the transcriptional repressor ID2, particularly affecting genes involved in cell migration and invasion [83]. Furthermore, redistribution of ER proteins has been observed in human GBM CRC tissues, supporting a broader role of this mechanism in tumor pathophysiology [12]. Analysis from The Cancer Genome Atlas (TCGA) revealed that elevated expression of key ERCYS regulators—DNAJB12, DNAJB14, and SGTA—is associated with poor prognosis across multiple malignancies, including head and neck squamous cell carcinoma, kidney renal clear cell carcinoma, colon adenocarcinoma, acute myeloid leukemia, adrenocortical carcinoma, uveal melanoma, pheochromocytoma and paraganglioma, and mesothelioma [12].

Chronic ER stress is an early and common feature of several neurodegenerative disorders, including Huntington’s. Alzheimer’s and Parkinson’s disease, and amyotrophic lateral sclerosis (ALS). These conditions are characterized by the accumulation of misfolded and aggregated proteins, which trigger proteotoxic stress responses. In Huntington’s disease, the aggregation of polyglutamine-expanded huntingtin (HTT-polyQ) is a key pathological feature, while in ALS, aggregation of mutant FUS (mutFUS) contributes to neuronal toxicity and disease progression. Recent studies have shown that full-length DNAJB12 and DNAJB14 cooperate to form a chaperone complex that suppresses mutFUS aggregation in an HSP70-dependent manner. This protective activity is lost in their naturally occurring truncated isoforms, which cannot form the complex. In contrast, while full-length DNAJB12 exacerbates HTT-polyQ aggregation, its short isoform mitigates aggregation and improves cellular outcomes. These findings underscore the isoform-specific and aggregate-type-dependent roles of DNAJB12 and DNAJB14, as well as their interactions with HSP70, as part of a dynamic chaperone network that regulates proteostasis in neurodegenerative disease [95].

In neuronal cells, the formation of mutant SOD1 inclusions is inhibited by PDI and ERp57, highlighting their protective roles in neurodegenerative settings [96–100]. Notably, the oxidoreductase activity of PDI, rather than its chaperone function, mediates protection against the cytoplasmic mislocalization of mutant TDP-43 in a redox-dependent manner [101]. Since both mutant SOD1 and TDP-43 predominantly localize to the cytoplasm, these findings suggest that PDI exerts its protective effects primarily through cytoplasmic mechanisms [102].

In the heart, ER stress is a key contributor to pathological processes such as ischemia–reperfusion injury, heart failure, and drug-induced cardiotoxicity. During these stress conditions, GRP78 has been shown to translocate to the plasma membrane, interacting with phosphoinositide 3-kinase (PI3K) and activating downstream AKT signaling. This interaction promotes cytoprotective and cardioprotective responses, thereby enhancing cellular survival under stress [103].

Vascular smooth muscle cell (VSMC) migration into the neointima plays a critical role in the pathogenesis of atherosclerosis and restenosis following vascular injury. Previous studies have demonstrated that platelet-derived growth factor (PDGF) stimulates the redistribution of PDI to the cytosol, coinciding with elevated reactive oxygen species production. Importantly, silencing of PDI significantly impairs PDGF-induced VSMC migration, underscoring its functional relevance. Within the cytosol, PDI interacts with RhoGDI1, Rac1, and RhoA in a PDGF-dependent manner, suggesting its involvement in Rho GTPase-mediated cytoskeletal remodeling. These findings support a model where PDI functions as a cytosolic regulator of PDGF-driven VSMC migration, acting through the Nox1-dependent redox system and downstream GTPase signaling pathways [104].

Finally, Recent findings indicate that viral infection induces a global and interorganellar spatial redistribution of proteins, with the ER exhibiting the most pronounced remodeling [21]. Among the affected proteins, AGR2/PDIA17 undergoes significant intra- and intercellular redistribution in response to viral challenges. This change alters the functional outcome of infected cells and neighboring immune cells, a process mediated through the redox-dependent interaction of AGR2 with cytosolic TRAF3 [66].

UDP-Glucose: Glycoprotein Glucosyltransferase 1 (UGGT1) is expelled from the ER under conditions of ER stress, a phenomenon that has functional implications during enterovirus A71 (EV-A71) infection. UGGT1 is redeployed to the cytosol, facilitating viral RNA synthesis [67]. This cytosolic relocalization mirrors aspects of the ER protein reflux process, in which ER-resident proteins exit the ER via stress-mediated mechanisms. The dual observation that UGGT1 exits the ER under stress [28] and subsequently acquires functional activity in the cytosol highlights a mechanism by which viruses may co-opt stress-induced protein trafficking pathways to support their replication (Fig. 1).

Overexpression of DNAJB12 and DNAJB14 has been shown to induce the formation of distinctive membranous structures within the nucleus, termed DJANGOS (DNAJ-associated nuclear globular structures) [90]. Similar structures containing DNAJB12 and DNAJB14 are also observed on the ER membrane, suggesting a potential link between these proteins and ER–nuclear membrane dynamics. Based on this observation, we speculate that DNAJB12/14 overexpression may promote ERCYS under cellular stress conditions. These DJANGOS structures are particularly interesting as they contain Hsc70, SGTA and markers of the ER lumen, nuclear membrane, and ER membrane [90], pointing to a complex interplay between chaperones and membrane systems in stress adaptation.

### Advances in the prediction and detection of protein mislocalization

Numerous tools have been developed to localize proteins within cells. Separation of subcellular organelles can be performed using detergents such as digitonin to extract the cytosol, followed by NP-40 to isolate membrane components, and subsequently stronger detergents for nuclear extraction [105]. Despite its simplicity, this method has limitations, as membrane composition may vary under different conditions, leading to increased sensitivity and membrane leakage [106]. These issues can be addressed by employing detergent-free fractionation methods, such as ultracentrifugation and differential centrifugation. These approaches rely on mechanical cell lysis and the separation is achieved based on size and density through sequential centrifugation at increasing speeds. Alternatively, sucrose gradient centrifugation separates organelles and other cellular components according to their density [107].

In contrast to these biochemical fractionation approaches, microscopy offers a complementary strategy for protein localization by directly visualizing proteins within intact cells. Accurate localization requires high-quality antibodies and the use of multiple antibodies to demonstrate colocalization of proteins in both the native and target organelles. Additionally, genetic manipulation is often employed to express the protein of interest fused to a fluorescent protein. This fusion may affect the subcellular localization of the protein due to inherent oligomerization tendencies of many commonly used fluorescent proteins. For example, EGFP has been shown to oligomerize, potentially hindering protein movement or affecting its function. Similar effects have been observed with other fluorescent proteins, such as tdTomato. Therefore, selecting appropriate monomeric fluorescent proteins is essential in assaying proteins functionality and localization [14, 108].

A substantial proportion of proteins lack reliable experimental data on their subcellular localization, underscoring the need for robust computational prediction methods. The sequence-based prediction approach make use of conserved and known amino acid sequences or/and sorting signals such as targeting and signal sequences. This method is simple and can applied for large dataset. Whereas prediction based on protein structure, functionality, and annotation, relies on information about functional domain and motifs, protein–protein interactions, and protein homology to other proteins [109, 110].

Although those methods can predict native proteins localization, they are unable to predict the localization of proteins outside their native organelles. Thus, a recently developed method was reported for this purpose. This methods relies on datasets of known proteins localization. Prediction of Unseen Proteins’ Subcellular localization (PUPS) combine cellular imaging models and protein language models utilizing protein sequence of known proteins and generalize this to unseen protein in unseen cell lines that do not exist in any of the existing atlases [111].

## Conclusions and future directions

The interorganellar spatial redistribution of proteins introduces an additional layer of complexity to eukaryotic cell biology, reflecting dynamic proteostasis that extends across multiple organelles. These spatial rearrangements are particularly pronounced in contexts such as cancer and viral infection, where proteome remodeling supports altered cellular states. However, the mechanisms governing protein redistribution, as well as the functional consequences of such interorganellar trafficking, remain largely unresolved. Elucidating the regulatory principles underlying protein relocalization may represent a major breakthrough in the field, as this phenomenon exemplifies how a single gene product can acquire distinct functions depending on its subcellular or extracellular localization.

ER-resident proteins can escape their canonical compartment and localize to non-ER locations, including the cytosol, nucleus, and extracellular space. Despite numerous reports of such “mislocalizations”, the mechanisms mediating ER protein escape remain poorly understood because they are often dismissed as experimental artifacts or contamination. However, accumulating data indicate that these proteins are not randomly wandering in the cell, but they acquire novel, context-specific functions following relocalization. Thus, mapping interorganellar spatial remodeling is essential for understanding different diseases and cellular stress responses. For instance, PDI—a prototypical ER-resident chaperone and isomerase—has been identified as a substrate of caspase-3 and caspase-7 when localized to the cytosol or nucleus [18]. Caspase-mediated cleavage of PDI in these compartments may occur via redox-dependent or redox-independent mechanisms, underscoring the protein’s functional versatility beyond its canonical ER role. Similarly, AGR2, which is synthesized and typically retained in the ER via a KTEL retention sequence, has been found in the extracellular space, where it can activate different signaling pathways [112, 113]. Furthermore, AGR2 can translocate to the cytosol, where it interacts with and inhibits pro-apoptotic signaling cascades. These examples highlight the biological significance of noncanonical protein localization in regulating cell fate. Importantly, such proteins are not mislocalized or lost; rather, they are intentionally redeployed to perform spatially distinct, pre-coded functions. This phenomenon reflects a previously underappreciated layer of functional proteome complexity.

Over the years, several mechanisms have been proposed to explain the escape of ER-resident proteins from their native compartment. These include ERAD evasion, ER membrane permeabilization, and chaperone-mediated transport processes. Despite these advances, the molecular diversity of triggers and the range of proteins undergoing reflux remain incompletely characterized. Further exploration of these pathways may reveal novel therapeutic strategies for diseases characterized by ER stress, such as chronic inflammatory disorders and various cancers. Moreover, investigating the signaling networks that regulate ER protein redistribution and identifying analogous escape mechanisms in other organelles will be essential for understanding the broader landscape of stress-responsive proteostasis. Particular attention should be directed toward elucidating the interplay between the UPR and ERCYS. Both pathways are believed to exert pro-survival and potentially pro-cancerous functions, and are frequently co-activated across various cancer types. Despite their mechanistic distinctions, UPR and ERCYS converge on a common goal: the reduction of ER substrate load under conditions of ER stress, suggesting a coordinated regulatory axis that may be critical for tumor progression and therapeutic resistance.

Despite advances in our understanding of subcellular organization, the molecular mechanisms governing protein redistribution and relocalization to the cytosol remain incompletely characterized. Elucidating the regulatory networks controlling global protein trafficking will be critical for the development of therapeutic interventions targeting a range of pathological conditions. In addition to defining these pathways, emerging technologies aimed at correcting protein mislocalization offer new therapeutic opportunities. Notably, a recent approach utilizing targeted relocalization-activating molecules (TRAMs) enables the restoration of proteins to their native compartments. By coupling TRAMs to shuttle proteins harboring organelle-specific targeting sequences, this strategy allows precise and efficient relocalization of mislocalized proteins to their appropriate subcellular destinations [114, 115].

## Data Availability

Not applicable.
